# Long-acting antiretroviral therapy in low-income and middle-income countries: considerations for roll-out

**DOI:** 10.1097/COH.0000000000000900

**Published:** 2024-11-11

**Authors:** Angela Tembo, Willem Daniel Francois Venter, Simiso Sokhela

**Affiliations:** Wits Ezintsha, Faculty of Health Sciences, University of the Witwatersrand, Johannesburg, South Africa

**Keywords:** considerations, long-acting antiretroviral therapy, low-income and middle-income countries, roll-out

## Abstract

**Purpose of review:**

Long-acting ART (LA-ART) in low-income and middle-income countries (LMICs) may address specific issues that affect people living with HIV (PWH) and people at substantial risk of HIV infection. We reviewed products in use and under consideration in LMICS, current and anticipated challenges for implementation, and offer strategies for effective rollout.

**Recent findings:**

Factors to consider for effective implementation of LA-ART in LMICs are: managing co-conditions (pregnancy) and comorbidities (TB and hepatitis B); medication access, both cost and supply-related; and health systems delivery mechanisms for products.

**Summary:**

LA-ART present promising new alternatives in LMICs. Although they may tackle certain adherence concerns and systemic issues, which impact delivery of services, significant obstacles remain before their widespread implementation in people that require them most, particularly in countries most affected by HIV. We offer best practices from prior experiences and implementation studies for effective coordination of multiple stakeholders, critical for effective rollout.

## INTRODUCTION

Current antiretroviral therapy (ART) for the treatment and prevention of the HIV are highly effective and well tolerated [1,2^▪^,^3^,^4^^▪▪^]. Oral integrase strand transfer inhibitor (INSTI)-based regimens – specifically tenofovir–lamivudine–dolutegravir (TLD) have markedly improved treatment outcomes and have become standard-of-care across the globe [5,6]. For prevention, oral tenofovir disoproxil fumarate (TDF) or tenofovir alafenamide in combination with lamivudine or emtricitabine (FTC) as preexposure prophylaxis (PrEP) reduces the risk of getting HIV by 99% if used effectively [7,8]. Equally, postexposure prophylaxis (PEP) significantly reduces the risk of getting HIV infection if initiated within 72 h of exposure [9,10^▪▪^].

Unfortunately, the daily oral ART requirement among people living with HIV (PWH) and for those on PrEP and PEP is a barrier to adherence [11,12], with reasons reported including pill burden, dosing frequency, duration of treatment, stigma, inadvertent disclosure and treatment interruptions related to changes in location of care [13,14^▪^,^15^]. Structural reasons cited include drug supply, service interruptions, and healthcare worker attitudes [13,16^▪^,17^▪^]. Nonadherence can worsen clinical outcomes, leading to increased mortality, persistent transmission, and increased drug resistance [18,19].

Long-acting ART (LA-ART) may address some of these adherence challenges related to daily dosing [20^▪▪^,^21^], with the potential to transform the HIV response in low-income and middle-income countries (LMICs) if used thoughtfully.

Long-acting therapeutics are already being used in the areas of contraception, harm reduction, diabetes, and mental health, with additional therapeutics under development for different conditions [22]. The range of technologies available includes various delivery systems for novel drug formulations such as transdermal patches, implants, depots, and intrauterine devices [18,22,23^▪^]. As momentum is gaining in the development of LA-ART, consideration of their integration in ART programmes in LMICs has become increasingly topical [24^▪▪^]. This review focuses on the agents approved by the Food and Drug Administration (FDA), or likely to be approved soon, that could be considered within LMICs, with discussion around current barriers, and strategies for the rollout of LA-ART. 

**Box 1:**
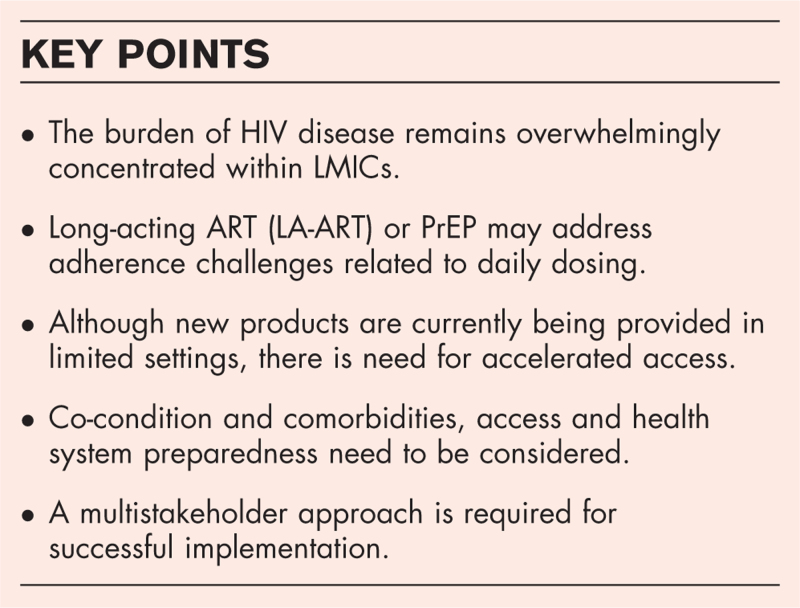
no caption available

## WHY LONG-ACTING ANTIRETROVIRAL THERAPY FOR LOW-INCOME AND MIDDLE-INCOME COUNTRIES?

The burden of HIV disease remains overwhelmingly concentrated within LMICs [25^▪▪^], and while the rollout of public health approach-guided antiretroviral programmes has been a significant success, recent indications have suggested that efforts to attain UNAIDS’ 95–95–95 targets have stalled [25^▪▪^].

The reasons for this are complex, but the implications are that an estimated 1.4 million infections will occur by 2030 because of the almost certain failure to reach the targets set for 2025 [26^▪▪^], again the majority occurring within LMICs. The consequences are significant, especially as all programmes report patients falling off and re-entering treatment, creating an ongoing burden on both health facilities for those who develop advances disease, as well as primary care programmes having to initiate the process of re-initiation, often with incomplete or completely absent prior treatment information.

Although mass access to LA-ART for treatment is unlikely to occur in the next few years, these formulations are being rolled out currently in demonstration projects for PrEP and could address many of the individual patient and systemic issues that pose challenges to UNAIDS targets. As additional agents become available, PrEP and even PEP offerings may strengthen and provide the platforms and experience to prepare for ART combinations with the same or similar medications in the near future.

## LONG-ACTION ANTIRETROVIRAL THERAPY LANDSCAPE IN LOW-INCOME AND MIDDLE-INCOME COUNTRIES

LA-ART agents registered by the US FDA and approved by various country regulatory authorities are relatively few, although many more are in the pipeline.

Long-acting cabotegravir (CAB LA) is a potent INSTI used for the treatment and prevention of HIV-1 infection [27] via a healthcare worker administered gluteal intramuscular injection [12]. The HIV Prevention Network (HPTN)-083 and HPTN-084 clinical trials, which included participants from LMICs, demonstrated 66 and 89% reduction in risks of HIV infection over daily oral TDF/FTC, respectively, when CAB LA was used for PrEP [23^▪^]. Modelling shows that intramuscular CAB can prevent three times more new infections than oral PrEP, with high acceptability in both high-income countries (HIC) and LMIC settings [28,29]. Despite strong evidence of its effectiveness in early 2020, and FDA approval in 2021, the introduction of CAB LA in LMICs has been very slow with very few countries approving its use [30]. Cost and production capacity remain a barrier to access [4^▪▪^]. In 2024, Malawi, Zambia, and Zimbabwe received PEPFAR-donated vials to boost their national PrEP programmes [31]. Availability in other LMICs is only through participation in implementation studies.

CAB LA in combination with long-acting rilpivirine (RPV LA) is as effective as existing oral ART in treating HIV-1 infection in virally suppressed adults and adolescent [23^▪^]. CAB/RPV is approved for monthly and 2-monthly dosing and is recommended in treatment guidelines for high-income countries as maintenance for people with virologic suppression [32]. Results from the Cabotegravir and Rilpivirine Long-Acting in Africa study (CARES), conducted in Kenya, South Africa, and Uganda, demonstrated noninferiority of the injectable combination in a switch study, when compared with oral therapy. Notably, the study necessarily excluded people with hepatitis B [33^▪▪^]. Importantly, however, demonstration projects have evaluated the utilization of long-acting regimens in PWH without virologic suppression and demonstrated the ability of long-acting CAB/RPV to achieve and maintain virologic suppression [34^▪▪^–36^▪▪^,37^▪^,^38^]. Subsequently, in the United States, the IAS-USA Guidelines Panel [39^▪▪^] and the DHHS *Guidelines for the Use of Antiretroviral Agents in Adults and Adolescents With HIV* committee [40^▪^] have now added long-acting cabotegravir with rilpivirine to their guidelines for those with virologic failure, adherence challenges to oral ART, and a high risk of HIV progression.

Long-acting lenacapavir (LEN LA) is a fusion HIV capsid inhibitor. The CAPELLA study examined lenacapavir in highly treatment-experienced participants and led to the drug's approval in the setting of multidrug-resistant HIV [41^▪▪^]. Other studies are underway to explore the efficacy of lenacapavir in various combination regimens as maintenance therapy for HIV, including one with daily oral bictegravir and another with weekly oral islatravir. In phase 3 of the PURPOSE 1 trial, the twice-a-year injection demonstrated 100% efficacy for HIV prevention in cisgender women [42^▪▪^]. Further results were recently presented from PURPOSE 2 (in cisgender MSM and transgender men and women), which showed 96% effectiveness in preventing HIV infection [43^▪▪^], which will support regulatory approvals. The pharmaceutical company that makes lenacapavir (Gilead) has stated a commitment to voluntarily license the medication to generic companies once approved for prevention [44^▪▪^].

## CO-CONDITIONS AND COMORBIDITIES

LMICs have a host of complex, geographically divergent needs that pose challenges to new long-acting agents, and these are often an after-thought for originator companies, where the US market is the primary focus, followed by European and other HIC populations.

One of the most complex populations is pregnant women with HIV, where issues for the women themselves (maintaining sufficient drug concentrations as volumes of distribution change markedly during pregnancy) to issues surrounding transmission and teratogenicity for the fetus, and breastmilk concentrations of the drug all need consideration. Experience with recent new antiretrovirals, including agents such as cabotegravir, has been reassuring, but safety issues with all new drugs, not just antiretrovirals, are often dealt with only after registration in HICs, meaning that reliance is placed on registries and observational data, leading to long delays before drugs can be recommended within public health programs [45^▪^]. This is of huge global consequence, as most people with HIV live within Southern Africa, and many of these are women of childbearing potential.

Many opportunistic infections require treatments with therapeutics that have drug–drug interactions with these long-acting agents A major concern is tuberculosis, highly prevalent in many LMICs as the most common serious opportunistic infection in people with HIV, principally because of the drug–drug interactions with the rifamycins. All current long-acting antiretroviral preparations are contraindicated with rifampin-based drugs, as significant reductions in drug levels occur with co-administration [46^▪^]. However, a strategy for all these opportunistic infections, including TB, may be to use oral integrase-inhibitor-based ART, potentially with dose-adjustment initially, with a switch to long-acting agents once the opportunistic infection is treated [1].

Hepatitis B is effectively treated with a combination of tenofovir prodrugs and cytosine analogues, components of all first-line oral antiretrovirals but not present in current long-acting formulations, a major consideration for public health programs where hepatitis B remains common, as most of these regions do not have hepatitis B vaccine programs. Consideration will need to be given to either developing hepatitis B screening programs if long-acting formulations are rolled out, and separate treatment strategies developed for those testing positive, including vaccination [47^▪▪^].

## ACCESS ISSUES

Access remains an issue with CAB LA where rollout has been slow. The availability of CAB for treatment studies and implementation research is restricted by the originator manufacturers, despite the voluntary licensing agreements with three generic companies signed in July 2022. The currently listed prices available for CAB LA per person per year are US$ 4 434  024 in the United States and US$ 3801.79 in the UK [48^▪▪^]. In South Africa, for example, the cost per CAB injection needs to be between $9.03 and $14.47 per injection for it to be as cost effective as TDF/FTC [29]. At these costs, CAB LA is unattainable for LMICs [49^▪▪^]. There is no indication currently as to the cost from the three generic companies, and indication that generic CAB LA will only be available from 2027 onwards. The Clinton Health Access Initiative has indicated that CAB LA, if volumes are attained, can be manufactured at costs near that of oral PrEP [50^▪▪^].

For CAB/RPV LA, the initial/loading dose is $5940, and maintenance injections are $3960 in the United States [51^▪^], a price tag that will hamper its widespread use in any LMIC settings. Furthermore, extra diagnostic costs need to be factored into the cost of CAB/RPV, currently calling for resistance and additional viral load testing, a consideration as many LMIC patients were exposed to efavirenz (a drug in the same class as rilpivirine) previously used as a first-line regimen in almost all LMICs [51^▪^]. Resistance testing is also recommended for late or missed injections [47^▪▪^] due to the long pharmacokinetic tail for CAB. Modelling suggests that to be cost-effective, CAB/RPV should target individuals who have sub-optimal adherence [47^▪▪^,^52^]. It is also unclear whether RPV LA will be available as a generic. Without inclusion in guidelines and volume guarantees, it seems unlikely that generic companies will invest in expensive manufacturing capabilities. Whether CAB/RPV will be used widely as treatment in LMICs remains to be seen [53^▪^].

## HEALTH SYSTEMS CHALLENGES

Initial long-acting agents are likely to increase the number of clinic visits, which conflicts with many donor and LMIC government efforts to decentralize care with ‘differentiated service delivery’ in the form of multimonth dispensing. Due to resource constraints, many people in LMICs receive their ART refills from lay providers, including peers and community health workers, within community settings. Often, these visits may only be once every 3–6 months, with a nurse consultation every 6 months [54]. CAB/RPV LA ART requires every 8-week injections, which means more visits and health worker time [51^▪^]. CAB/RPV injections need to be given by a trained healthcare worker and are not as simple as other intramuscular injections to administer. Restructuring of clinic visits would need to be considered.

Healthcare worker training and drug storage will also need to be factored in. LA-ART are administered intramuscularly, specifically in the buttock. CAB/RPV administration is technical; it administered using the ‘z-track’ method, which involves positioning an intramuscular injection into the gluteal muscles to reach the appropriate tissue layer, requiring a special needle for people with an elevated BMI [51^▪^]. Additionally, the recommended storage range for rilpivirine injection is 2° to 8°C (35.6° to 46.4°F) [55^▪^], making it harder to provide in mobile clinics and many community health facilities without refrigerators, especially in areas that have an intermittent electricity supply.

## LESSONS FROM PRIOR EXPERIENCE AND IMPLEMENTATION STUDIES

The future of LA-ART in LMICs is promising but requires concerted efforts for swift and effective implementation. The successful rollout of dolutegravir (DTG) has shown it is possible to overcome financial and structural barriers in the scale-up of innovative products [56^▪▪^]. In 2019, the WHO recommended the use of DTG as the preferred treatment for all populations. Collaborative partnerships between governments, pharmaceutical companies, and research institutions led to guideline recommendations and the groundbreaking pricing agreement, which reduced the cost of DTG by 75% in 2020 [56^▪▪^] Through collaborative efforts from community advocacy groups plus market-shaping interventions, the generic version of DTG is now in use by over 25 million PLHV in LMICs at less than US$45 per person per year. CAB/RPV will likely be the first LA-ART available in LMICs, though it is unlikely to be affordable at scale or anytime soon, even in generic form. A similar coordinated approach is required to track research project results and real-world lessons for guideline development.

There are also important lessons from existing HIV treatment and prevention programmes. Despite its efficacy, very few people are on oral PrEP. Only 1,9 million people in LMICs received oral PrEP at least once in 2022 [4^▪▪^]. PrEP provision in most countries is largely led by professional nurses who are certified to provide HIV treatment and care, limiting who can initiate PrEP, and where people can access it [57^▪^]. In July 2022, the WHO released guidance to simplify the delivery of oral PrEP. De-medicalizing PrEP can increase access and enable a wider range of providers to prescribe and dispense PrEP [58^▪^]. Task shifting can enable the use of different staff cadres to administer injections (both for treatment and prevention), subject to receiving the necessary training. Additionally, settings such as pharmacies, instrumental in the COVID-19 response, can be used as injection sites.

However, there is still much to be learned about the provision of person-centered healthcare in the HIV field. Current PrEP offerings in countries likes South Africa, Zimbabwe, Zambia, and Malawi include a choice of oral (TDF/3TC), injectable (CAB LA), and vaginal products (dapivirine ring) tailored to lifestyles but only in demonstration projects. ART demonstration studies would provide more insights into factors that influence movement between products. For instance, the SEARCH study just performed an intervention providing people needing PrEP in Kenya and Uganda, a choice of oral PrEP versus intramuscular CAB for PrEP, demonstrating a 56.4% increase in biomedical prevention coverage with the provision of long-acting PrEP. The conclusion of this study and others demonstrate that offering people the choice of HIV biomedical prevention options, including LA PrEP, can increase prevention coverage and reduce the incidence of HIV.

## CONCLUSION

Long-acting antiretrovirals offer exciting new options within LMICs. However, while they may address some adherence challenges and perhaps even systems issues LMICs face, there are enormous hurdles to face before we see their widespread use in the populations that most need them, and in countries most affected by HIV. The most immediate and pressing problem to solve is the current patent, cost, and manufacturing stranglehold originators have around the medications that is holding back the long and difficult work that needs to be done on figuring out how to implement these complex medications within LMIC health systems.

## Acknowledgements

*None.*

### Financial support and sponsorship

*This work was supported, in whole or in part, by the Bill & Melinda Gates Foundation (INV-037064). The conclusions and opinions expressed in this work are those of the author(s) alone and shall not be attributed to the Foundation. Under the grant conditions of the Foundation, a Creative Commons Attribution 4.0 License has already been assigned to the Author-Accepted Manuscript version that might arise from this submission. Please note works submitted as a preprint have not undergone a peer review process.*

### Conflicts of interest

*W.D.F.V.'s unit (Ezintsha) receives funding for conducting studies from the Bill and Melinda Gates Foundation, SA Medical Research Council, National Institutes for Health, Unitaid, Foundation for Innovative New Diagnostics (FIND), Merck Sharp & Dohme (MSD), Janssen and the Children's Investment Fund Foundation (CIFF); has previously received funding from USAID; and receives drug donations from ViiV Healthcare, MSD, and Gilead Sciences for investigator-led clinical studies. W.D.F.V. receives honoraria for educational talks and advisory board membership for Gilead, ViiV, Mylan/Viatris, Merck, Adcock-Ingram, Aspen, Abbott, Roche, J&J, Sanofi and Virology Education. SS receives honoraria for educational talks from Janssen, MSD, ViiV and advisory board participations from ABBVIE.*

## REFERENCES AND RECOMMENDED READING

Papers of particular interest, published within the annual period of review, have been highlighted as:

▪ of special interest
▪▪ of outstanding interest
